# Measuring anomalies in cigarette sales using official data from Spanish provinces: Are the anomalies detected by the Empty Pack Surveys (EPSs) used by Transnational Tobacco Companies (TTCs) the only anomalies?

**DOI:** 10.18332/tid/143321

**Published:** 2021-12-15

**Authors:** Pedro Cadahia, Antonio Golpe, Juan M. Martín-Álvarez, Eva Asensio

**Affiliations:** 1Department of Economics, University of Huelva, Huelva, Spain; 2Department of Quantitative Methods for Economics and Business, Universidad Internacional de La Rioja, Logroño, Spain

**Keywords:** empty pack survey, cigarette sales, illicit trade, machine learning

## Abstract

**INTRODUCTION:**

There is a literature that questions the veracity of the studies commissioned by transnational tobacco companies (TTCs) to measure the illicit tobacco trade. Furthermore, there are studies that have indicated that the empty pack surveys (EPSs) ordered by TTCs overestimate the size of this trade. This study simultaneously analyzed whether the EPSs established in each of the 47 Spanish provinces were accurate and measured anomalies observed in provinces where sales exceed expected values.

**METHODS:**

To achieve the objectives of this study, provincial data on cigarette sales, prices and GDP per capita were used. These data were modeled with machine learning techniques that are widely used to detect anomalies in other areas.

**RESULTS:**

The magnitude of the average anomaly in provinces where sales are higher than their expected values exceeds 40%, while the average anomaly in provinces where sales are lower than their expected values (as detected by the EPSs) is <15%. Furthermore, the results reveal that there is a clear geographical pattern to the provinces in which sales below reasonable values are observed. In addition, the values provided by the EPSs in Spain, as indicated in the previous literature, are slightly overestimated. Finally, some regions bordering other countries or that are highly influenced by tourism have observed sales that are higher than their expected values.

**CONCLUSIONS:**

Cooperation between countries in their tobacco control policies can have better effects than policies developed based on information from a single country. The lack of control over the transactions of tourists and the inhabitants of bordering countries can cause important anomalies that distort the understanding of tobacco consumption that governments have based on official data.

## INTRODUCTION

Some theoretical and empirical works have questioned the Empty Pack Surveys (EPSs) because they are commissioned by transnational tobacco companies (TTCs), and their methodology and validity are not certain^1^. The EPSs consist, fundamentally, of collecting empty packages deposited on the ground or in city bins and checking what rate of them are not legal or domestic products. This context of the non-independence of the EPSs has generated a multitude of articles that have analyzed the relationship between what the TTCs show regarding the illicit tobacco trade (ITT) and the official data published by the government. In addition to the EPSs, the TTCs make reports, usually annually, about the ITT. In this vein, some studies have concluded that the reports made by TTCs require greater transparency, greater external scrutiny, and the use of independent data^2^. Another issue criticized by some studies is the funding and dissemination of ITT research by TTCs through corporate social responsibility initiatives. In this context, the literature has concluded that if TTC data on the ITT cannot meet the standards of accuracy and transparency established by high-quality research publications, a solution may be to tax the TTCs and provide the resulting funds to experts independent of the tobacco industry, using previously developed reliable models to measure the ITT^3,4^.

In this context of non-independence, many studies have proposed methodologies, using official data, to measure the size of the illicit tobacco market^5^. In this part of the literature, many results have been achieved. Some studies have concluded that industry-funded estimates inflate the likely levels of illicit cigarette use^6,7^. Others indicate that industry warnings against tax increases in certain countries based on illicit trade rates are not justified^8-10^.

Given this academic trend that doubts the design and implementation of the surveys commissioned by the TTCs, there are many studies that have focused on analyzing how illicit trade impacts the health of the population, as well as the policies implemented by the governments. Some studies have suggested that tobacco tax policies to control the prevalence of smoking and reduce national healthcare expenditures should be accompanied by a greater effort to curb smuggling activities across borders^11^. On the other hand, some studies have analyzed the impact of plain tobacco packaging on illicit trade^12,13^. Finally, there are some studies that have carried out a type of EPS parallel to those commissioned by TTCs to verify their veracity^14,15^.

Although there are many studies that have made an effort to contrast the EPSs with official data or to conduct parallel surveys, all have focused on analyzing whether the data provided by the TTCs regarding the rates of illicit trade are true. However, a recent study has indicated that the actual smoking prevalence sometimes does not match the estimated actual consumption derived from aggregated data on official sales^16^. Furthermore, there are border areas in which the prevalence of smoking is underestimated because official data do not consider what smokers buy in areas with an attractive price differential. In the same way, other work has also indicated that the excessive production of cigarettes suggests a possible excess supply of cigarettes in some countries, probably diverted towards illicit trade^17^. Studies that question the veracity of EPSs focus on verifying whether the rates of illicit trade in a given country are real. However, what is indicated in the works cited in this paragraph highlights the need to also study the excess sales rates of products at the borders of certain countries, which can then be sold illegally in other countries with an attractive price differential.

**Figure 1:**
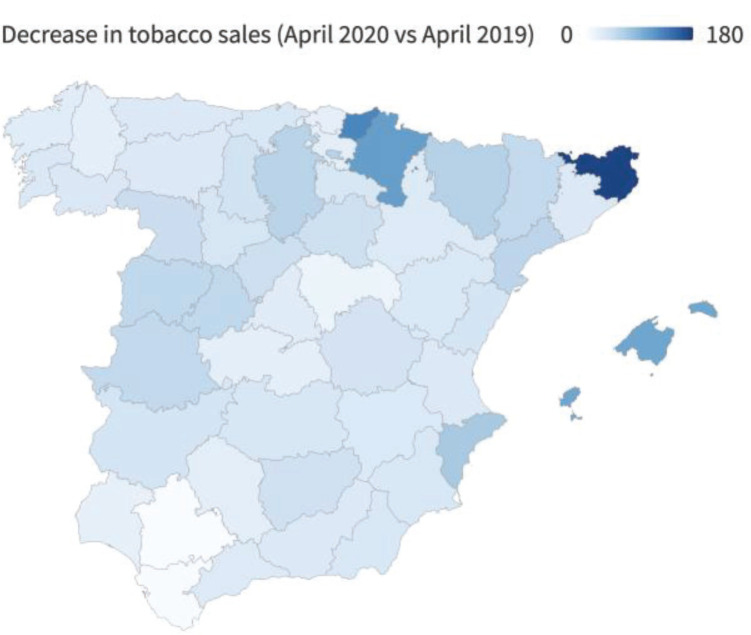
Percentage of year-on-year drop in tobacco sales (April 2019 to April 2020)

Thus, to simultaneously study the veracity of EPSs and excess sales in areas that border another country with a higher tobacco price, it is necessary to study a country that has borders with countries that have lower and higher prices. Spain shares a border with France and Gibraltar, two countries with which it maintains a price differential by excess and by deficit. In addition, some recent studies have indicated that distortions are observed around the Spanish borders with France and Gibraltar^18,19^. Although the cited study indicates that there are distortions in the provinces bordering France and Gibraltar, it is important to know the magnitude of these distortions. The current health crisis caused by COVID-19 caused the borders in Spain to close during April 2020, and the effect on cigarette sales is shown in Figure 1. In some provinces, sales decreased by up to 180 percent, while in other provinces, tobacco sales did not decline. Therefore, focusing this study on Spain seems reasonable if we want to simultaneously analyze the veracity of EPSs and excess sales in border areas.

In this context, our study analyzed the two components of the anomalies in official tobacco sales, that is, both the provinces in which official sales are lower than expected and those in which sales are higher than their expected value. There is no evidence in the literature that clarifies the territorial anomalies that are observed in both directions. To the best of our knowledge, this study is the first to analyze, simultaneously, whether the provisions of the EPSs are fulfilled, contrasting it with official data and, furthermore, which provinces have sales that are above reasonable values. In this context, this study sets two objectives: 1) to check whether the EPS overestimates illicit trade, and 2) to estimate anomalies that cause tobacco exits from Spain to other countries where prices are higher than in Spain.

## METHODS

### Data

Our empirical analysis was developed using a panel of data from the Spanish provinces from 2002 to 2017, the year when the latest data on provincial GDP were published. For cigarette consumption, we used the official annual tobacco sales and the average price of a pack of 20 cigarettes in euros, as published by the Commission for the Trade of Tobacco. The real gross domestic product (GDP) is available from the National Institute of Statistics in Spain. All series employed here are per capita (for those aged ≥18 years) and expressed in real terms using the consumer price index (CPI base 2016). The descriptive statistics of the dataset are shown in Table 1.

**Table 1:** Descriptive statistics of the data used

*Province*	*Years*	Per capita cigarette sales*					Price*					Per capita GDP*				
		*Mean*	*SD*	*Quartile*			*Mean*	*SD*	*Quartile*			*Mean*	*SD*	*Quartile*		
				*Q1*	*Q2*	*Q3*			*Q1*	*Q2*	*Q3*			*Q1*	*Q2*	*Q3*
Albacete	16	93.45	25.84	64.12	101.18	115.86	3.07	1.19	1.93	2.93	4.39	19.72	3.34	18.01	21.27	21.47
Alicante	16	135.80	55.05	78.74	127.64	184.19	3.10	1.15	2.02	2.93	4.36	19.75	2.21	19.37	20.19	21.01
Almería	16	114.20	38.21	72.19	118.10	146.96	3.14	1.17	2.03	2.98	4.41	21.59	2.59	20.89	21.92	23.40
Álava	16	81.18	22.34	56.98	84.07	101.80	3.06	1.21	1.92	2.91	4.37	36.26	6.96	32.92	38.29	40.87
Asturias	16	88.74	21.88	64.93	95.44	107.41	3.06	1.19	1.91	2.90	4.31	20.90	3.56	19.37	22.41	23.12
Ávila	16	93.47	25.53	65.35	99.97	115.41	3.08	1.20	1.94	2.92	4.37	18.93	3.27	16.96	20.30	21.09
Badajoz	16	98.68	28.85	66.72	110.11	123.38	3.04	1.20	1.87	2.91	4.29	17.53	2.96	16.05	18.74	19.34
Islas Baleares	16	168.33	72.05	95.93	150.77	228.62	3.13	1.15	2.05	2.93	4.37	27.29	3.66	26.06	28.11	29.15
Barcelona	16	87.59	27.11	58.16	89.84	109.03	3.04	1.20	1.89	2.86	4.34	28.75	5.25	26.19	30.21	31.28
Vizcaya	16	77.93	18.21	58.77	81.43	92.03	3.06	1.21	1.92	2.91	4.37	28.92	5.62	25.51	30.90	31.83
Burgos	16	87.44	23.79	61.89	91.81	109.79	3.05	1.21	1.89	2.88	4.33	26.61	4.62	24.13	28.33	29.22
Cáceres	16	99.28	26.80	69.04	109.19	121.22	3.04	1.19	1.89	2.90	4.34	17.25	3.14	15.54	18.33	18.86
Cádiz	16	75.60	33.69	37.81	82.92	107.68	3.04	1.19	1.89	2.90	4.32	18.82	2.40	18.64	19.65	20.27
Cantabria	16	93.99	30.05	65.44	102.02	118.87	3.08	1.20	1.93	2.91	4.41	22.51	3.54	20.86	23.99	24.55
Castellón	16	102.53	33.67	66.20	103.11	133.72	3.07	1.17	1.94	2.92	4.36	25.61	3.68	24.76	26.19	27.02
Ciudad Real	16	96.62	26.99	66.04	105.98	120.09	3.06	1.20	1.91	2.91	4.37	20.66	3.31	19.17	21.80	22.65
Córdoba	16	88.33	32.20	50.46	98.48	115.96	3.03	1.21	1.88	2.88	4.35	17.91	2.97	16.83	18.99	19.45
La Coruña	16	81.50	20.58	58.94	87.07	98.53	3.05	1.20	1.89	2.89	4.35	21.68	4.30	19.17	23.58	23.98
Cuenca	16	98.42	25.96	68.50	106.47	121.25	3.08	1.20	1.94	2.93	4.42	20.53	3.97	18.54	21.77	22.65
Guipúzcoa	16	146.87	51.79	95.44	144.96	193.39	3.03	1.21	1.85	2.86	4.32	31.07	5.47	28.36	33.18	33.92
Gerona	16	267.40	97.35	169.15	261.90	358.11	3.05	1.20	1.87	2.86	4.36	29.22	4.39	27.97	30.58	31.27
Granada	16	99.06	31.07	63.37	105.28	126.55	3.08	1.19	1.94	2.93	4.38	18.28	2.97	17.18	19.32	20.06
Guadalajara	16	93.49	28.64	61.79	96.66	116.66	3.08	1.18	1.97	2.92	4.38	20.93	2.69	20.53	21.98	22.47
Huelva	16	113.75	41.13	65.68	125.89	150.66	3.04	1.19	1.88	2.89	4.31	19.40	2.70	18.84	19.91	21.07
Huesca	16	116.51	33.40	79.16	124.19	147.17	3.07	1.20	1.92	2.90	4.35	26.90	5.23	23.35	28.87	30.21
Jaén	16	95.73	27.76	64.02	106.05	118.55	3.05	1.19	1.91	2.93	4.31	17.70	2.77	16.33	18.62	19.38
León	16	84.99	21.14	61.87	92.17	103.14	3.08	1.21	1.92	2.91	4.35	19.93	3.27	18.45	21.61	21.93
Lleida	16	140.99	52.46	85.33	144.39	188.71	3.02	1.20	1.84	2.84	4.34	29.59	4.90	26.65	31.15	33.19
Lugo	16	73.08	15.41	56.07	78.52	87.09	3.06	1.20	1.92	2.89	4.37	20.07	4.33	17.87	20.89	22.89
Madrid	16	88.05	27.11	59.71	90.11	108.47	3.07	1.19	1.93	2.91	4.35	33.79	5.86	30.85	35.65	36.87
Málaga	16	113.73	50.43	60.59	114.07	160.94	3.10	1.17	2.01	2.92	4.34	19.02	2.68	18.81	20.02	20.43
Murcia	16	107.57	32.88	71.37	111.70	136.50	3.09	1.17	1.99	2.94	4.35	21.52	3.36	20.33	22.57	23.18
Navarra	16	139.86	40.06	97.19	148.17	174.95	3.05	1.19	1.89	2.88	4.29	30.88	4.81	28.90	32.48	33.60
Orense	16	73.21	14.59	57.04	80.84	86.36	3.07	1.20	1.92	2.90	4.38	18.59	3.54	16.47	19.68	20.82
Palencia	16	89.41	23.05	64.70	96.01	108.03	3.07	1.20	1.92	2.90	4.35	24.18	4.14	21.85	25.57	26.43
Pontevedra	16	78.35	21.73	53.76	84.15	99.32	3.05	1.19	1.89	2.89	4.36	20.62	3.67	19.11	21.86	22.74
La Rioja	16	87.69	22.32	63.63	90.91	106.82	3.06	1.19	1.93	2.90	4.37	26.56	4.36	24.49	28.18	28.94
Salamanca	16	84.75	24.26	57.80	94.72	106.90	3.08	1.20	1.92	2.92	4.29	19.64	2.85	18.45	20.62	20.98
Segovia	16	86.75	25.42	58.20	91.45	109.43	3.08	1.20	1.93	2.91	4.36	22.86	3.11	22.15	23.92	24.44
Sevilla	16	86.20	38.24	42.25	95.66	120.93	3.05	1.19	1.90	2.90	4.28	20.76	3.22	19.61	22.07	22.61
Soria	16	83.82	20.09	61.58	89.94	99.13	3.11	1.20	1.96	2.96	4.39	23.91	3.88	21.42	25.48	26.35
Tarragona	16	115.17	41.05	71.29	112.86	153.18	3.11	1.16	2.01	2.94	4.40	30.05	4.15	27.91	31.13	31.66
Teruel	16	89.73	21.70	65.42	95.51	108.95	3.09	1.21	1.95	2.94	4.40	25.17	3.99	23.31	26.90	27.96
Toledo	16	97.11	30.58	62.88	103.27	124.87	3.07	1.18	1.94	2.90	4.35	19.55	2.65	19.44	20.30	20.93
Valencia	16	98.39	31.02	64.17	102.72	123.52	3.01	1.18	1.88	2.86	4.30	23.34	3.54	21.68	24.57	25.53
Valladolid	16	85.01	24.65	58.01	89.36	105.15	3.05	1.20	1.90	2.88	4.36	24.50	4.12	22.49	26.03	26.62
Zamora	16	79.91	19.41	58.84	87.73	95.88	3.06	1.20	1.91	2.89	4.33	18.27	3.48	16.13	19.31	20.69
Zaragoza	16	94.87	26.92	64.64	99.35	118.11	3.05	1.18	1.91	2.90	4.35	26.60	4.46	24.66	28.25	29.05

* Per capita sales are measured in packs of 20 cigarettes per year. The price is measured in real euros of 2016. GDP per capita is expressed in thousands of real euros of 2016. SD: standard deviation. Q1,Q2, Q3 are the 25, 50 (median) and 75% quartiles.

### Empirical methodology

Data-driven anomaly detection systems have been discussed in the literature as distortion detection systems in many fields of application^20-23^. Such systems aim to detect any abnormal deviations from the normal observations in any given data set. Therefore, these methodologies provide a good opportunity to detect anomalies in tobacco sales. Furthermore, given its abovementioned characteristics, Spain seems to be a reasonable candidate country in which to quantify anomalies.

The aim of this work is to detect tobacco sales anomaly at the provincial level. The prediction of the upper and lower limits of tobacco sales at the provincial level is proposed as a methodology to identify any abnormal deviations in tobacco sales behavior. The proposed methodology is a supervised learning method, which adjusts the model of the relationship between tobacco sales (the dependent variable) and price and GDP (the independent variables). On the other hand, the detection and estimation of anomalies is performed through an unsupervised method, as mentioned before, by means of the computation of upper and lower intervals. Several statistical and machine learning models were compared to find the best model for predicting the tobacco sales in each province (these methods/models are presented in this section).

The main methodology consists of splitting the data into training and test sets for all the available provinces in the Spanish territory, where the training set consists of all the provinces available with the exception of the province to be predicted, which is in the test set. In other words, all provinces are used to predict the sales in a given province without including that province. As is commonly used to explain tobacco consumption behavior in Spain^24-26^, the dependent variable is per capita tobacco sales for each province, and the independent variables are per capita GDP and price:

*Tobacco sales = f(price, GDP, Pop* ^18+^ *)*

To model the relationship between the dependent variable and the independent variables (the characteristic vector *x*), two supervised learning methods have been used. In addition, to estimate the upper and lower limits of the prediction interval, quantile predictions are used to generate intervals following the methods explained in this section.

The first method used to model the relationship between the variables is the quantile regression (QR) method. This method was introduced^27^ for the estimation of models in which the quantiles of the response variable are modeled to depend on the independent variables. The τth quantile for a population is the sample where the 100/τ percent proportion of the population lies. This model of the relationships between different quantile predictors and the dependent variable, in this case, provides clear interpretability for the anomaly detection results, as it is possible to identify an anomaly within a given range^28^.

The conditional α-quantile *q* of a scalar variable *Y* is *P(Y*≤*q|I*)=α, where the probability 0<α<1 is given and I denotes an information set generated by independent variables *X*. A complete justification of this method is given elsewhere^27^.

For the purposes of this work, two models were combined to build the intervals for detecting anomalies. That is, the conditional 0.1-quantile was set as a lower bound and the 0.9-quantile as an upper bound for each province. By construction, the probability that a value belongs to the interval between the upper and lower bounds is:

*P(l ≤ X ≤ u) = P(X ≤ u) - P(X ≤ l) = 0.9 - 0.1 = 0.8*

In contrast to ordinary least squares, which estimates the conditional mean, this method is based primarily on choosing a model for estimating the conditional quantile. Depending on the strength of the assumptions imposed, a range of parametric or nonparametric options are available^29^.

For assessing the models, the conditional median response (the 0.5 quantile) for each province was estimated. Not only are the models evaluated for the quality of their punctual predictions, but the ability of their intervals to select the best model with good performance in both tasks is also evaluated, which is discussed later in this section.

In this work, a bagging method is proposed to estimate the conditional quantiles. A combination of random forest (RF) and QR was proposed^30^, resulting in the quantile regression forest (QRF) approach. One of the main differences between RF and QRF is that in QRF, each node of each tree maintains the values of all observations in that node, but RF only maintains the mean of the observations found in the node^30^. Ranger is a high-speed implementation of RF or recursive partitioning^31,32^ and is particularly well-suited for high-dimensional data^a^.

To detect anomalies, two methods were selected to build the prediction intervals (PIs) through conditional quantiles; for every new observation of the response variable, there is a high probability that it lies within the PI^24^. Furthermore, an anomaly detection and quantification system is proposed that uses an upper bound and lower bound computed using the fitted models.

As mentioned before, the PIs are computed using the calculation of the chosen conditional 0.1-quantile and 0.9-quantile for the lower bound and upper bound, respectively. One requirement for the choice of α for the intervals is the use of a symmetric range (i.e. one cannot use the 0.1 quantile as the lower bound and the 0.7 quantile as the upper). It is not of interest for this study to find the best intervals within a model but to provide a methodology for computing the share of abnormalities, as shown in this section.

The proposed method assumes a uniform distribution with endpoints at the lower and upper limits of the computed PIs. Every point outside this interval range is considered abnormal, and the intervals are also used to quantify the share of abnormalities in the response variable. Specifically, the observed response for province p is abnormal if either case is true:

*y_i_ < y_t_*

or

*y_i_ > y_(100-t)_*

where *η* (0 ≤ t ≤ 100%) represents the chosen quantile level given that the interval is symmetrical, and the limits *y*(*100-t*) percent and *y(t)* percent represent the upper and lower conditional quantiles, respectively. The choice of a smaller value of t will lead to a larger number of provinces being predicted to be abnormal.

To train the model, a data partition was performed, as explained in the above sections, and the predictive accuracy of the models was measured by splitting the data into training and test sets.

The error assessment was performed either by using a 0.5-quantile prediction for the quantile analyses or the interval prediction was used to determine the magnitude of the abnormality of tobacco sales that were evaluated with the results of surveys, and certain metrics were used to assess the quality of these intervals.

The performance of the predicted responses (*ŷ_i_*) in relation to the observed responses (*y_i_*) of the training and test sets were assessed by computing the error metrics given in the Supplementary file.

In addition to evaluating the punctual predictions, the estimated prediction interval has also been evaluated in this work. The academic literature has placed a special emphasis on point prediction relative to interval predictions and predictive densities; consequently, there has been little work on the evaluation of PIs^33^.

A review of the methods for evaluating point predictions used in this work is presented in the Supplementary file, where the selected metrics assess

the accuracy of the training and test sets. However, as the main idea in forecasting is to decrease uncertainty, an interval prediction evaluation is performed as well. Supplementary file Table 3 summarizes the metrics used to evaluate the prediction interval.

Although anomaly detection has been used in many previous works in other disciplines, the novelty of this method is that it complements abnormality detection with abnormality quantification. As explained previously, the computed intervals are used to quantify the share of abnormalities, and Table 2 shows the formulas that are applied to carry out this task.

**Table 2:** Quantification of anomalies in per capita tobacco consumption

*Anomaly ratio*	*Calculation way*
Upper anomaly ratio (UAR)	$\text{UAR}=\frac{y_{i}-p_{u}}{p_{u}}$
Lower anomaly ratio (LAR)	$\text{LAR}=\frac{y_{i}-p_{l}}{p_{l}}$

Where p_l_ is the lower and p_u_ the upper prediction interval (PI).

As the final product of this work, the model should be able to discern between a province with abnormal tobacco sales and a province without abnormal tobacco sales but also, when an abnormality is detected, it should also be able to determine whether this abnormality is due to a quantity below the lower bound or to a quantity above the upper bound.

## RESULTS

The results are shown in three parts. First, the evolution in the anomalies detected over time is shown (for both the provinces in which sales are lower than the expected values and those in which sales are higher than expected). Second, the temporal evolution of the regional anomalies detected in Spain is shown. Finally, the geographical distribution of the detected anomalies is shown.

### National temporal evolution of the anomalies detected

**Figure 2:**
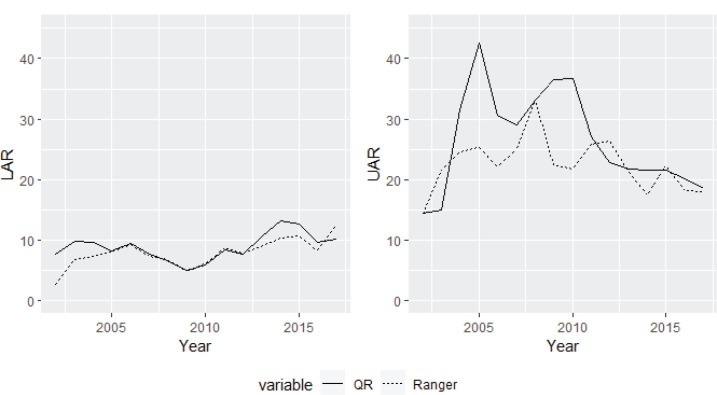
Average UAR and LAR for the Spanish territory

As indicated, to quantify the anomalies in the Spanish provinces, we use the upper anomaly ratio (UAR) and the lower anomaly ratio (LAR). The average UAR and LAR for the Spanish territories are calculated by averaging the ratio of tobacco sales per capita observed to be below the lower limit (the lower prediction bound) and the ratio of tobacco sales per capita observed to be above the upper bound (the upper prediction bound). Figure 2 shows the aforementioned index, and the answers to two important questions can be observed: 1) the magnitude of the average upper anomaly exceeds 40%, while the average lower anomaly is <15%; and 2) the lower anomalies are trending upward over time and the upper anomalies are trending downward.

Regarding the magnitude of the LAR and UAR, although, as will be seen in this section, geographical patterns play a key role, it seems that there is a significant difference between the indices. The average LAR represents the average percentage of those provinces that have observed sales below the estimated values. Therefore, given that this index is conditioned by cross-border and illegal trade, it seems that both activities have remained constant during the period studied. However, the average UAR increased notably until 2005 and has decreased since then. The UAR index represents the average anomaly in the provinces in which the observed sales exceed those estimated by the model. For this reason, both the effect of tourism and that of the cross-border trade with countries where tobacco is more expensive than in Spain determine the magnitude of the UAR index. The provinces that border France have been considered to exhibit a cross-border effect, while the rest of the provinces are highly influenced by tourism. Supplementary file Figure 1 shows the part of the average UAR that represents cross-border effects and the part that represents tourism effects. Both effects seem to be decreasing, with the trend in the cross-border effect being stronger. This last highlight is consistent with recent evidence on cross-border transactions between Spain and France^13^.

### Regional temporal evolution of the anomalies detected

**Figure 3:**
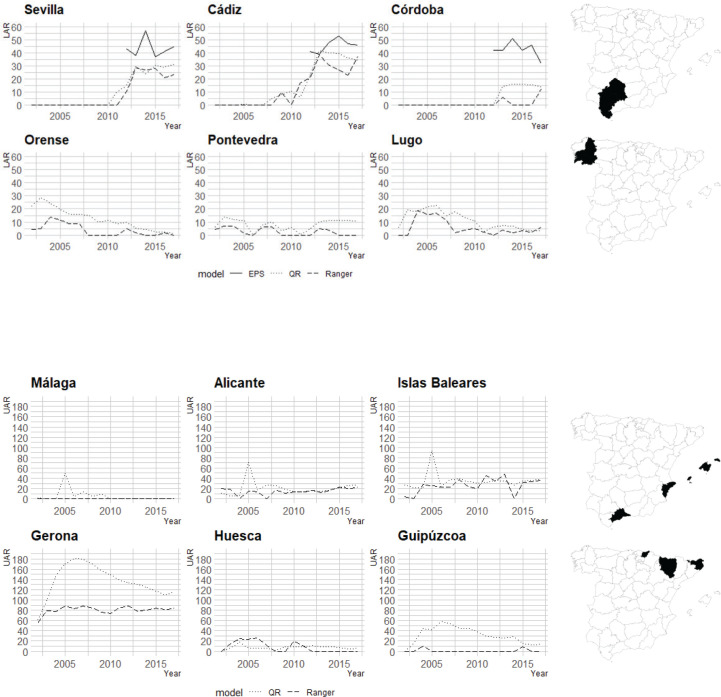
Temporal evolution of UAR and LAR in the Spanish provinces

Once the magnitudes have been estimated at the national level, it is interesting to analyze the temporal evolution of the UAR and LAR in the Spanish provinces. First, regarding the LAR, there are 6 provinces that stand out for their behavior. As shown in Figure 3, there are three provinces in southern Spain (Sevilla, Cádiz and Córdoba) in which the LAR is estimated for the first time in 2010 and grows notably until 2017, reaching values close to 40% in some cases. On the other hand, in three other provinces (Orense, Pontevedra and Lugo), the LAR is decreasing; in addition, it rarely takes values close to 20%. In addition, Figure 3 also shows the values given by the EPSs that were conducted by the TTCs from 2012 to 2017. As the figure shows, in line with the previous literature, it seems that the use of independent data provides estimates of the illicit market that are lower than those provided by the EPSs. Specifically, in Cádiz, the EPS is slightly overestimated, and in Córdoba, there are substantial differences.

Six provinces also stand out in the temporal evolution of the UAR in the Spanish territory. On the one hand, Malaga, Alicante and Islas Baleares provinces that are highly influenced by tourism^a^ present a similar trend. On the other hand, in the provinces that border France, such as Gerona, Huesca and Guipúzcoa, the UAR exhibits a decreasing trend, with the anomalies detected in Gerona being of a much higher magnitude. As indicated in the introduction, Gerona is the Spanish region in which sales fell the most due to border closures related to the public health crisis of COVID-19. Therefore, these results are consistent with what the raw data indicated.

### Regional distribution of the anomalies detected

**Figure 4:**
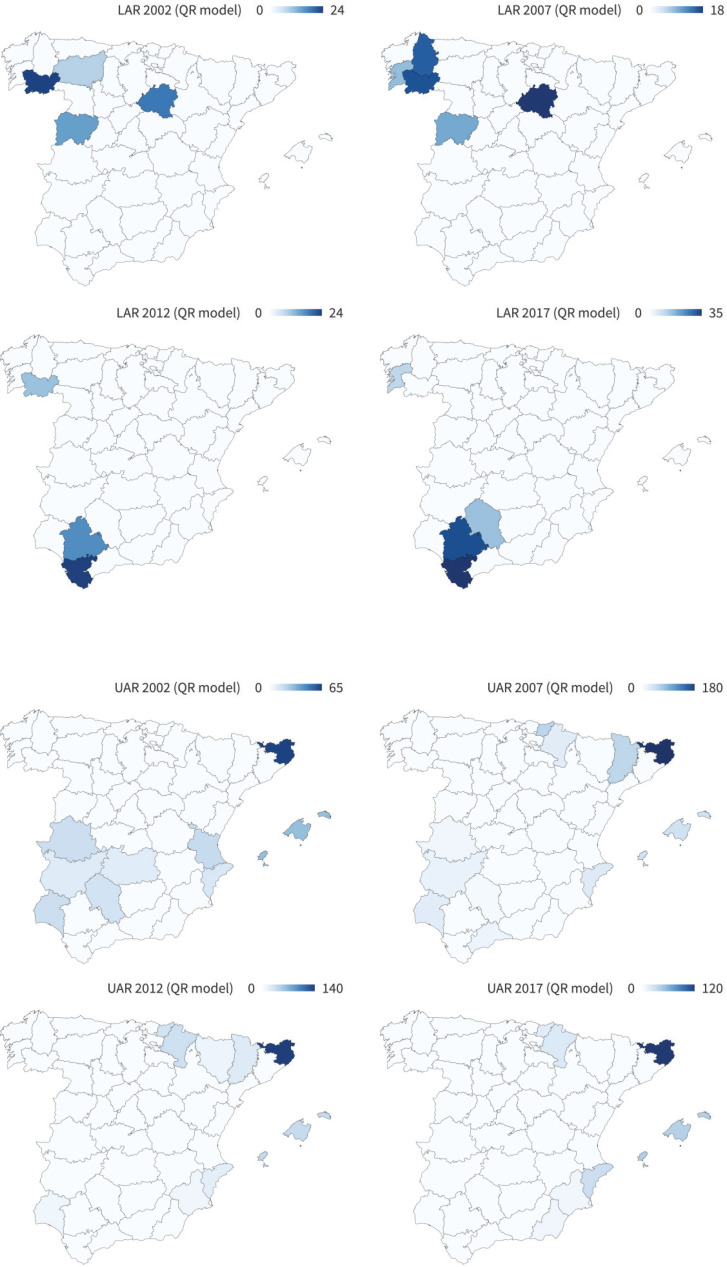
Geographical distribution of LAR and UAR in the Spanish provinces (QR model)

Although the provincial evolution of the UAR and LAR provides important information, the geographical distribution of the anomalies helps us to understand the contagion effect of the UAR and the LAR, as well as tobacco consumption behavior at the borders with other countries. In this sense, Figure 4 shows the geographical distribution of the LAR and UAR according to the QR model, respectively. As can be seen, the anomalies in the provinces in which lower-than-estimated sales have been observed reach 35% and are concentrated in the Northwest in 2002 and in the South in 2017, which is consistent with the previous literature. In addition, regarding the UAR, the anomalies in the provinces with sales above those estimated reach values of 190%. Finally, while these high UAR values were concentrated in tourist provinces and provinces bordering Portugal in 2002, in 2017 the concentration in tourist provinces remained the same, but it is now the border with France where the anomalies are located.

One of the main contributions of the present study is the quantification of the LAR and UAR. Along these lines, the closure of Spain’s borders with other countries due to the health crisis caused by COVID-19 during the months of April and May 2020 has made it possible to analyze the robustness of the results of this work. As shown in the Supplementary file Figures 2 and 3, both the geographical pattern and the magnitude of the UAR estimated in this work are robust to the decreases in tobacco sales in Spain in the months of April and May 2020. On the one hand, tobacco sales fell in April and May by up to 180% and 160%, respectively. This magnitude is consistent with the anomalies shown in Figure 4. Furthermore, the geographical patterns are also consistent, with the greatest decreases in tobacco sales being observed in the areas near the border with France and in the tourist provinces. In addition, the areas near the border with Gibraltar are those in which tobacco sales have decreased the least during the border closures. It is precisely in these provinces that the highest UARs are observed in the latest years analyzed.

## DISCUSSION

In recent years, there has been growing interest in knowing the mechanisms that can control cigarette consumption due to the great impact of tobacco consumption on public health. Along these lines, due to the free movement of people and to illegal activities, legal tobacco sales are sometimes not a faithful representation of tobacco consumption. Specifically, in some provinces legal tobacco sales are not a good indicator of consumption (see the case of Sevilla, Cádiz, Córdoba, Guipúzcoa, Gerona or Huesca). To address this, multiple studies have been commissioned by the TTCs to demonstrate that there is illicit cross-border activity that generates greater consumption of tobacco within the population than governments believe. Although there are many initiatives commissioned by TTCs, EPSs are the most widespread. These EPSs are meant to detect what appears to be illegal trade in provinces where there is less than reasonable tobacco consumption. This study has shown, in line with the previous literature, that in Spain, the EPSs that are performed to estimate the size of the illicit market (mainly in the border areas with Gibraltar) overestimate its size. In addition, as a contribution to the literature, in this work, anomalies have been detected in provinces where sales are higher than their expected values, information that TTCs ignore.

To the best of our knowledge, this study is the first to quantify anomalies in regional tobacco sales in Spain, including anomalies in provinces where more than the expected values are sold. In particular, the results found for the provinces in which observed tobacco sales are below expected values are similar to those found in previous literature: the EPSs overestimate the value of illicit trade. As can be seen, over time the provinces in which sales below fair values are observed go from the north to the south of Spain. This could indicate that the agents operating illegally have changed their route or origin of the illegal tobacco, from Portugal to Gibraltar. On the other hand, the provinces in which sales above fair values are observed are currently concentrated on the border with France, which could be motivated by the price differential. In reference to the anomalies detected in provinces in which tobacco sales above reasonable values are observed – something that is rarely found in the literature – the findings are novel.

Specifically, as Figure 1 shows, the provinces where tobacco sales are highest relative to the expected values are those where sales have fallen the most due to border closures driven by the COVID-19 public health crisis. This finding undoubtedly confirms that tobacco sales in Spain are conditioned by the effects of tourism and the price differentials with France and Gibraltar. Furthermore, cross-border tobacco purchases between Spain and France have been decreasing in recent years, somewhat in line with the findings in previous literature. Although cross-border tobacco purchases between Spain and France have decreased in recent years, the deviation in sales in the provinces where more tobacco is sold than is reasonable (tourist provinces and those on the border with France) is much higher on average than the deviations in those provinces where sales are below the expected values (those on the border with Gibraltar). This result is novel, given that the anomalies in tourist provinces and provinces that border France had never been quantified. Therefore, when the TTCs present the results of the EPSs in Spain, the opposite effect of what the EPSs detect must be taken into account and considered by the government.

Our results indicate the provinces in which smoking control policies cannot be evaluated using official sales, since these sales are inaccurate. In this sense, we find that the provinces in which sales are most affected are in border and tourist areas, evidencing the existence of large-scale illegal trade and cross-border purchases. The results suggest that for some years, there have been no anomalies in areas that border Portugal. Therefore, the results reveal the effectiveness of the common policies implemented by the governments of Spain and Portugal, which consist of maintaining a low-price differential between the countries. The results seem to support the need for border countries with free movement to harmonize the fiscal structure of tobacco products. Progress is being made on issues such as product traceability to combat illicit trade. However, it appears that adjusting the price differential is a determining factor in reducing incentives for illicit trade.

All these results provide insights for the agendas of academics and governments. The academic community should bear in mind that there is more evidence about the overestimation of illicit trade in EPSs and that the average of the excess anomalies is much higher than the average of the deficit anomalies. In addition, policy makers should consider that there are provinces where the effectiveness of anti-smoking policies cannot be evaluated using official sales. The allocation of resources to control smoking must take into account the abnormalities identified in this study. If not, the provinces in which there are excessive consumption distortions will receive more resources to control a tobacco habit that is not real. In contrast, the border provinces with Gibraltar will have fewer resources to control smoking if official sales are used, when the reality is that there is hidden consumption due to illicit trade.

### Limitations

The results shown are not without limitations. On the one hand, cigarette sales have been used. Although cigarettes account for almost 90% of total tobacco consumption in Spain, part of the anomalies detected may be motivated by the consumption of substitute products. In addition, the use of aggregated data prevents knowing individual behaviors. By using individual data, it could be known whether individual sociodemographic characteristics are influencing the abnormalities detected. Another line of future research consists of separating which part of the detected anomalies come from illegal activities. Finally, the used method assumes that tobacco control measures have the same compliance rate in all the provinces studied.

## CONCLUSIONS

The results seem to show that EPSs overestimate the value of illicit tobacco trade. Furthermore, in Spain, the provinces with sales volumes above the expected values have higher ratios than those in which sales are below the expected values. Therefore, it seems that the sum of the effect of tourism and cross-border purchases between Spain and France is higher than the cross-border purchases between Spain and Gibraltar detected by the EPS. Finally, these anomalies prevent the Spanish government from knowing the total benefit to public health generated by policies against smoking.

## Data Availability

The data supporting this research are available from the authors on reasonable request.

## Footnotes

a The R libraries ranger was used to fit a QRF model, respectively with the default settings.
