# Supplementary material for: Measuring anomalies in cigarette sales using official data from Spanish provinces: Are the anomalies detected by the Empty Pack Surveys (EPSs) used by Transnational Tobacco Companies (TTCs) the only anomalies?
Source: Tob Induc Dis. 2021 Dec 15;19:98. doi: 10.18332/tid/143321 (PMC8669702; doi:10.18332/tid/143321)
Supplement: Supplementary file 1 [file TID-19-98-s1.pdf]

Supplementary Material

Supplementary Table 1. Averaged error metrics for the fitted models at the training set.

Averaged error metric	Calculation way
Prediction error	$e_i = y_i - \hat{y}_i$
Mean squared error	$MSE = \frac{1}{n}\sum_{i=1}^{n} e_i^2$
Mean absolute error	$MAE = \frac{1}{n}\sum_{i=1}^{n} \lvert e_i \rvert$
Mean absolute percentage error	$MAPE = \frac{1}{n}\sum_{i=1}^{n} \lvert \frac{e_i}{y_i} \rvert$
Median Absolute Error	$MedAE(y, \hat{y}) = median(\lvert y_i - \hat{y}_i \rvert, \dots, \lvert y_n - \hat{y}_n \rvert)$
Median Squared Error	$MedAE(y, \hat{y}) = median((y_1 - \hat{y}_1)^2, \dots, (y_n - \hat{y}_n)^2)$
Median Absolute Percentage Error	$MedAPE(y, \hat{y}) = median\ of\ sorted\ \lvert \frac{\hat{y}_i - y_i}{y_i} \rvert$

Note:

* It is computed by ordering the absolute percentage error (APE) from the smallest to the largest and using its middle value (or the average of the middle two values if N is an even number) as the median.

* For further understanding of this formulas and its statistical properties, (Hyndman & Koehler, 2006).

Supplementary Table 2. Prediction interval accuracy for the fitted models at the training set.

Accuracy metric	Calculation way
Mean Internal Score	$MIS = (p_u - p_l) + \frac{2}{\alpha}(p_l - y)1(y < p_l) + \frac{2}{\alpha}(y - p_u)1(y > p_l)$
Range	$range = \frac{1}{n}\sum_{i=1}^{n} \vert\frac{p_{u_i}}{p_{l_i}}\vert$
Coverage	$covareage = \frac{1}{n}\sum_{i=1}^{n}\left(1(y_i < p_{l_i}) \times 1(y_i < p_{l_i})\right)$
Pinball	$pinball = (1-\alpha)\sum_{\hat{y}_i<\hat{b}_i} \vert\hat{y}_i - \hat{b}_i\vert + \alpha \sum_{\hat{y}_i<\hat{b}_i} \vert\hat{y}_i - \hat{b}_i\vert$

Note:

*Where pl is the lower PI, pu the upper PI, α is the significance level, y the actual value and 1(·) is the indicator function, for more details see[30] .

*Where n is the number of sample size, p_u for upper PI, p_l for lower PI and y the actual value for each observation *i*.

*Where $\hat{b}_i$ is the predicted value of a interval (either an upper, or a lower).

*MIS balance *coverage* and *range* of the PI, the best choice is when a model has high coverage, but also short intervals.

*Pinball loss function show how well a quantile capture the data, the lower the value of pinball is, the closer the interval is to the specific quantile of the holdout distribution.(Koenker, 2005).

MAE	MSE	MAPE	MEDAE	MEDSE	MEDAPE	MAE
QR	16,3	1185,9	0,13	8,4	85,19	0,09
Ranger	17,79	1029,36	0,16	10,27	117,78	0,11

Supplementary Table 3. Averaged Error metrics for the fitted models at the training set.

Model	MAE	MSE	MAPE	MEDAE	MEDSE	MEDAPE
QR	17,8	1292,18	0,15	17,8	1292,18	0,15
Ranger	17,75	1027,63	0,16	17,75	1027,63	0,16

Supplementary Table 4. Average metrics for the prediction at the test set.

Model	MIS	Coverage	Range	Pinball_lw	Pinball_hi
QR	116,81	25,04	20,14	2702,23	6269,05
Ranger	99,41	48,52	32,22	2328,82	5305,85

Supplementary Table 5. Averaged Interval metrics for the predicted intervals.

Supplementary Figure 1. Touristic and crossborder UAR in Spain.

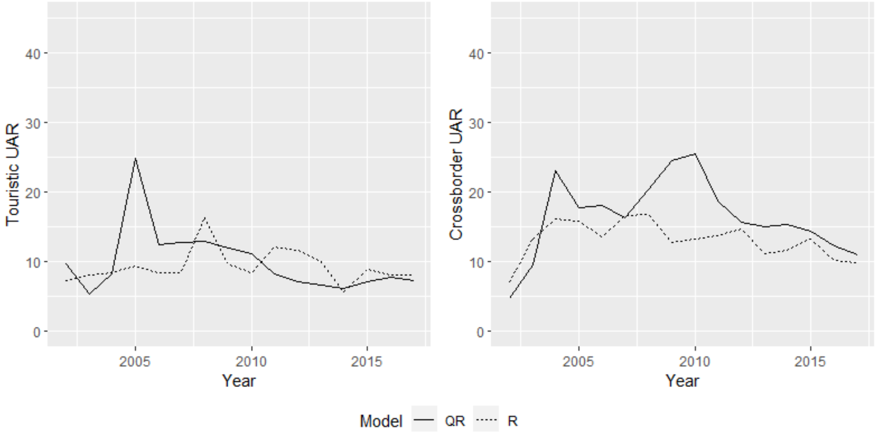

Supplementary Figure 2. Comparison between the results of the model and the fall in sales of April 2020

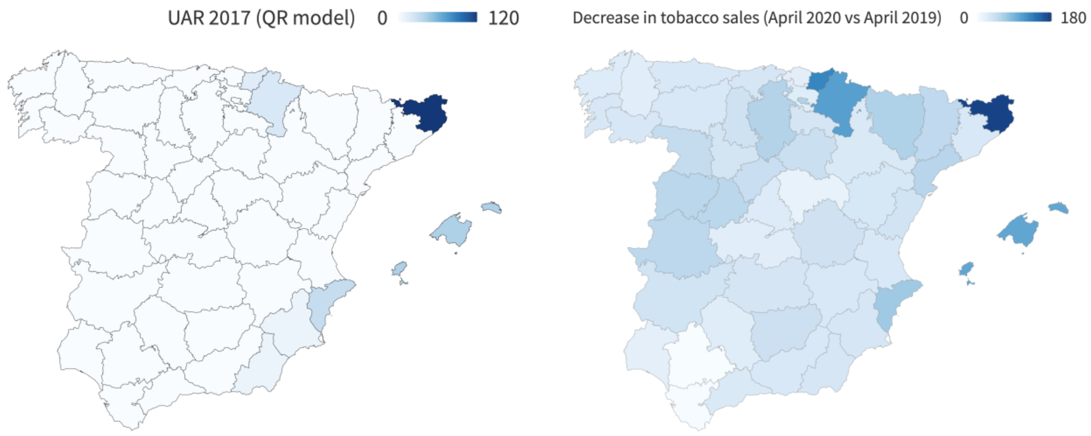

.

Supplementary Figure 3. Comparison between the results of the model and the fall in sales of May 2020.

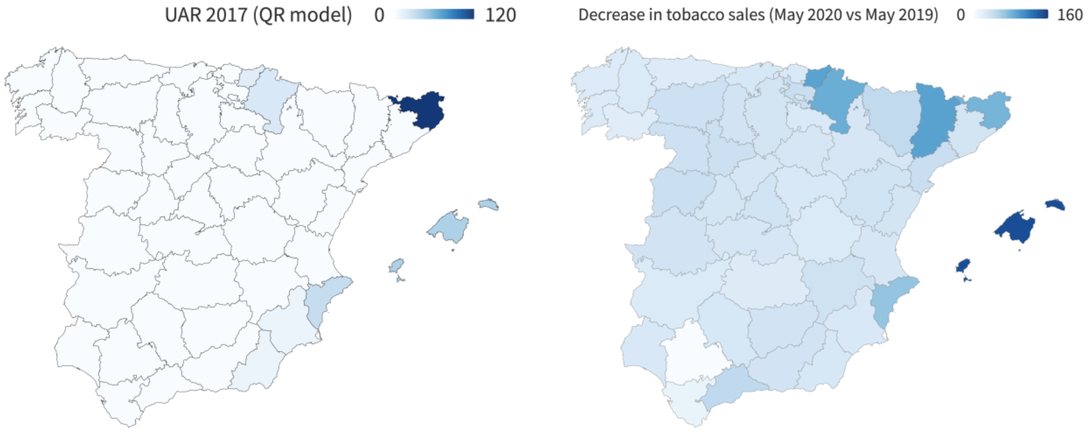

Training set error

The training set comprises the data without the province to predict for every year, at the following Supplementary Figure 4, it shows the averaged results (Table 5).

Supplementary Figure 4. Scatter plots for the error of fitted models at the training set.

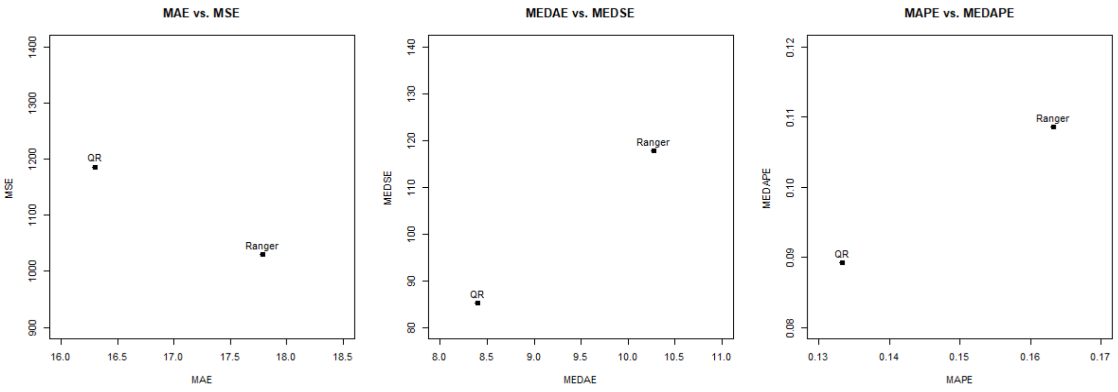

As discussed on some research of error measurement different statistical properties reflects every metric, with the errors shown on the previous table QR shows minimum error on training set.

In order to avoid the bias of the averaged metrics, the following density plots are shown in the Supplementary Figure 5. with this we can confirm the superiority of QR over Ranger at the training set.

Supplementary Figure 5. Density plots for the errors of fitted models at the training set.

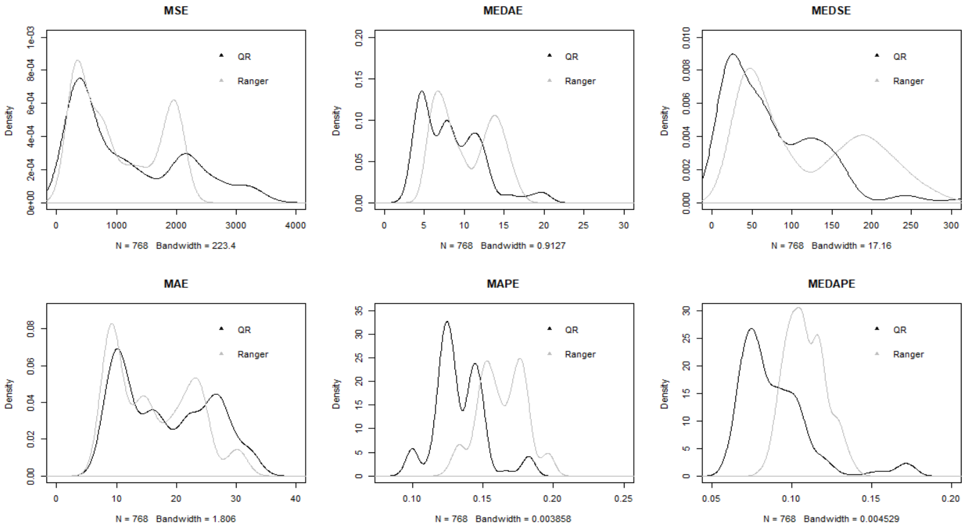

Test set error.

The test set comprises the data with the province to predict for every year, at the Supplementary Figure 6, it shows the averaged results of the metrics presented on subsection 2.2 are shown.

Supplementary Figure 6. Scatter plots for the fitted models at the training set.

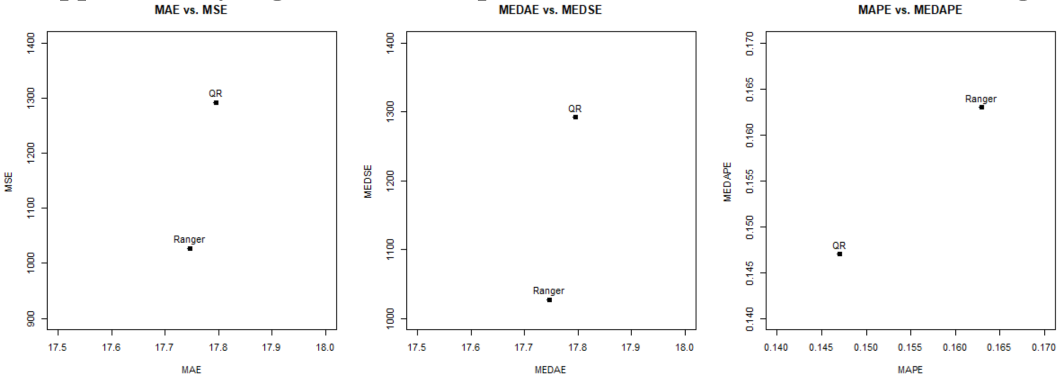

At the test set the predictions errors are very similar, but the square metrics (MSE and MEDSE) penalizes the QR showing a slightly superiority of Ranger.

The density plots shows the distribution of errors at the test set (Supplementary Figure 7), where the similarity of test errors are present with the difference at the MAE Error.

Supplementary Figure 7. Density plots for errors of the fitted models at the test set.

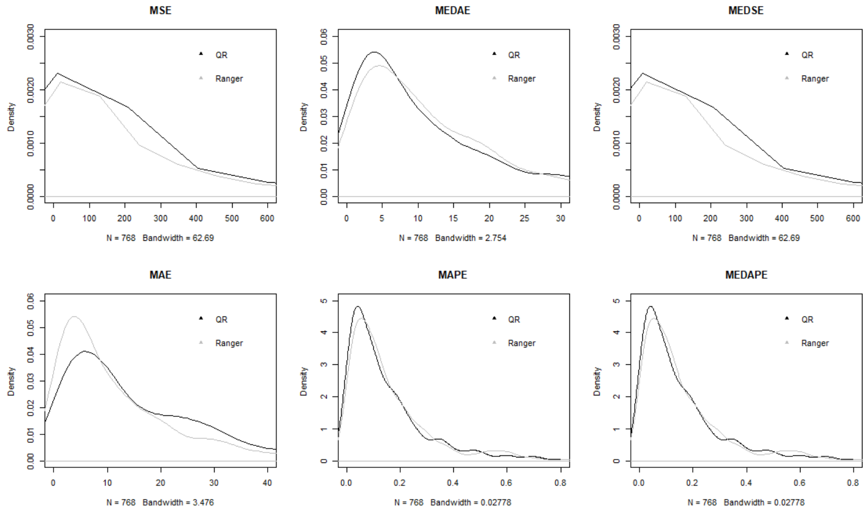

Interval Score Metrics.

This subsection shows at metrics for assessing the prediction intervals which are the main novelty use for this work. By using these metrics, a wide overview of how intervals are fitted is potentially used to discard a method for abnormality detection and quantification as this work propose.

The results are shown visually at Supplementary Figure 8, where the superiority of QR over Ranger is present. The MIS and Pinball (average Pinball lw and Pinball hi) shows better performance of the intervals, also the pinball score for bot metrics. The Range and coverage of Ranger are smaller than QR, in this case is the intervals are wide enough to cover the regular points and having a better fit of QR intervals.

Supplementary Figure 8. Averaged Interval metrics for the predicted intervals.

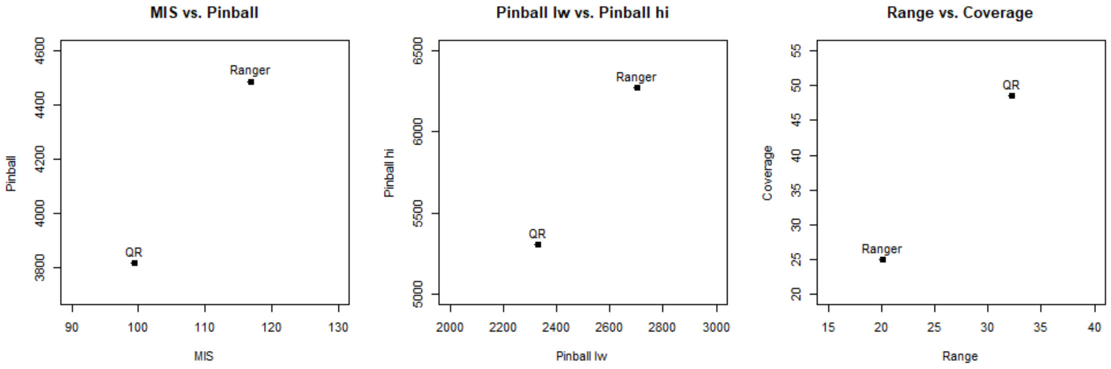

© 2021 Cadahia P. et al.
